# Closed-Loop Frontal Midlineθ Neurofeedback: A Novel Approach for Training Focused-Attention Meditation

**DOI:** 10.3389/fnhum.2020.00246

**Published:** 2020-06-30

**Authors:** Tracy Brandmeyer, Arnaud Delorme

**Affiliations:** ^1^Osher Center for Integrative Medicine, School of Medicine, University of California, San Francisco, San Francisco, CA, United States; ^2^Centre de Recherche Cerveau et Cognition (CerCo), Université Paul Sabatier, Toulouse, France; ^3^CNRS, UMR 5549, Toulouse, France; ^4^Swartz Center for Computational Neuroscience, Institute of Neural Computation, University of California, San Diego, La Jolla, CA, United States

**Keywords:** adaptive neurofeedback, frontal mid-line theta, meditation, EEG, attention training

## Abstract

Cortical oscillations serve as an index of both sensory and cognitive processes and represent one of the most promising candidates for training and targeting the top-down mechanisms underlying executive functions. Research findings suggest that theta (θ) oscillations (3–7 Hz) recorded over frontal-midline electrodes are broadly associated with a number of higher-order cognitive processes and may serve as the mechanistic backbone for cognitive control. Frontal-midline theta (FMθ) oscillations have also been shown to inversely correlate with activity in the default mode network (DMN), a network in the brain linked to spontaneous thought processes such as mind-wandering and rumination. In line with these findings, we previously observed increased FMθ oscillations in expert meditation practitioners during reported periods of focused-attention meditation practice when compared to periods of mind-wandering. In an effort to narrow the explanatory gap by directly connecting observed neurophysiological activity in the brain to the phenomenological nature of reported experience, we designed a methodologically novel and adaptive neurofeedback protocol with the aim of modulating FMθ while having meditation novice participants implement breath-focus strategies derived from focused-attention mediation practices. Participants who received eight sessions of the adaptive FMθ-meditation neurofeedback protocol were able to significantly modulate FMθ over frontal electrodes using focused-attention meditation strategies relative to their baseline by the end of the training and demonstrated significantly faster reaction times on correct trials during the n-back working memory task assessed before and after the FMθ-meditation neurofeedback protocol. No significant differences in frontal theta activity or behavior were observed in the active control participants who received age and gender matched sham neurofeedback. These findings help lay the groundwork for the development of brain training protocols and neurofeedback applications that aim to train features of the mental states and traits associated with focused-attention meditation.

## Introduction

Insights into the nature of cortical oscillations reveal the unique capacity for humans to voluntarily control and interact with their own neural activity when presented with real-time sensory feedback (Sitaram et al., 2017). Neurofeedback research dates back to the early 1960’s, with findings providing preliminary evidence for successfully modulated neural activity through sensory feedback based on event-related potentials (ERPs) and spectral power (Kamiya, 1968). Recent advancements in the development of neurofeedback protocols that implement sophisticated and source specific methodologies have contributed to the resurgence of neurofeedback applications and clinical interventions, as well as to the expanding prominence and popularity of various forms of brain training (Owen et al., 2010; Rabipour and Raz, 2012). With mounting interest and potential for neurofeedback applications to successfully modulate the cognitive processes underlying attention and emotion regulation (Anguera et al., 2013; Sitaram et al., 2017), innovative approaches that adapt and individualize the nature of the feedback in real-time require further scientific study.

Recent findings suggest that individuals with impaired attentional control engage in maladaptive emotion regulation strategies (i.e., rumination, compulsive thought processes) and are rendered more prone to the risk of depression and anxiety disorders (DeJong et al., 2019). The default mode network (DMN) is a large and distributed network comprised of the posterior cingulate cortex (PCC), medial prefrontal cortex (mPFC), medial and lateral temporal lobes, superior and inferior frontal gyri, and the posterior inferior parietal lobule (Gusnard et al., 2001; Raichle, 2015). The DMN shows consistent activation during various forms of self-generated thought, including *spontaneous thought processes* such as mind-wandering, creative thinking, day dreaming, planning, as well as more maladaptive forms of self-generated thought such as rumination and compulsive thought processes (Mason et al., 2007; Hasenkamp and Barsalou, 2012; Kucyi et al., 2013; Fox et al., 2015, 2018). Practices such as meditation have recently been shown to engage brain structures and networks directly implicated in the regulation and focusing of attention, presumably through the active regulation and cultivation of an awareness of the occurrence of spontaneous thought processes (awareness of the when the mind wanders away from the object of focus during meditation (i.e., the breath) being the primary ‘objective’ of focused attention meditation; Brandmeyer et al., 2019). Interestingly, research conducted on long-term expert meditation practitioners found greater reductions in DMN activity during meditation practice than during other types of attention-demanding tasks (Garrison et al., 2015).

Electroencephalography (EEG) findings from our previous research (Brandmeyer and Delorme, 2018) found that increased cortical frontal midline theta oscillations (FMθ; FCz, Fz; 4–6 Hz) were present during internally guided states of focused-attention meditation when compared to reported periods of mind wandering and spontaneous thought. These electrophysiological findings are consistent with previous meditation research that measured focused-attention during meditation (Aftanas and Golocheikine, 2001; Kerr et al., 2013). Increases in FMθ and mid-frontal θ (Cz) have been repeatedly observed during tasks that assess measures of executive function such working memory and conflict detection (Bollimunta et al., 2011; Cavanagh and Frank, 2014; Enriquez-Geppert et al., 2014; Cavanagh and Shackman, 2015). Together these findings suggest a functional relationship between the sources contributing to broader mid-frontal θ activity and the maintenance of top-down representations of goal states, learning, directed attention, and the regulation of spontaneous thought (Cavanagh and Frank, 2014; deBettencourt et al., 2015). Furthermore, FMθ activity has been shown to inversely correlate with the blood-oxygen-level-dependent (BOLD) signal in the DMN (Scheeringa et al., 2008), suggesting that these broad and distributed networks may functionally compete for resources. We hypothesize that the role of FMθ observed during focused-attention meditation practice is likely to result from the goal of (1) sustained attention (most often focus is on the breath) during focused-attention meditation, (2) the need to detect when the mind has wandered, and (3) the need to redirect attention back to the object of focus. This cycle, in effect, strengthens the top-down processes involved not only in the focusing of attention, but in the active monitoring of mental sates, while falling in line with the established literature regarding the specific role of FMθ in learning (Swick and Turken, 2002; Haegens et al., 2010). Cavanagh and Frank (2014) have suggested that broader frontal θ oscillations may serve as a candidate mechanism by which neurons communicate top-down control over long range and broad networks. Broader frontal θ oscillations have been proposed to function as a temporal template for organizing mid-frontal neuronal processes (Cavanagh and Frank, 2014), with theta-band phase dynamics thought to entrain disparate neural systems when cognitive control is needed (e.g., through entrainment of cortical and subcortical areas via the cingulate cortex; Bollimunta et al., 2009; Morecraft and Tanji, 2009).

Research findings suggest that the given size of a functional brain network may determine its oscillatory frequency, therefore the larger and more distributed the network, the slower the underlying oscillation (von Stein and Sarnthein, 2000). Electrophysiology findings from research on learning, memory, feedback, feedback-driven learning (Kahana et al., 2001; Marco-Pallares et al., 2008; van de Vijver et al., 2011), as well as broader cognitive control processes (Cavanagh et al., 2012) provide convincing evidence that theta band oscillatory activity may serve as the underlying “language” of the prefrontal cortex for local and network wide communication (Cavanagh and Frank, 2014; Cohen, 2014). This likely reflects the role of the intrinsic architecture and structure of the prefrontal cortex in supporting the rhythmogenesis of theta-band activity, and that specific and local neural computations are what account for fluctuations in EEG (Cohen, 2014). The functional implication of these findings suggests that broader frontal theta oscillations may provide a framework for adjusting and monitoring temporally sequenced activity, functioning as a hub for theta phase-synchronized information transfer (Cohen and Cavanagh, 2011; Cavanagh and Frank, 2014; Cohen, 2014). Additional empirical research findings suggest that the neural mechanisms underlying sustained attention heavily rely on FMθ dynamics such as phase synchronization resulting in greater measures of power (Friese et al., 2016; Wei et al., 2017). Frontal theta oscillations may therefore serve as an ideal candidate for neurofeedback protocols aimed at training and improving cognitive functions such as sustained and focused-attention, with possible transference to cognitive faculties that fall under the broader umbrella of executive functions (Enriquez-Geppert et al., 2014).

Interestingly, many conditions that see improved measures of behavioral outcomes associated with regular meditation practice are consistent with the conditions that improve in response to neurofeedback training (Brandmeyer and Delorme, 2013, 2018). Theoretically, both methods involve training specific mental states and neural measures of cognitive processes underlying attention and emotion regulation, with more long lasting traits and skills developing cumulatively over time. Research findings suggest that both ADHD patients and individuals diagnosed with depression benefit from meditation training (Evans et al., 2018) as well as neurofeedback training protocols (Arns et al., 2009; Peeters et al., 2014). Thus, the early stages of mental training in focused-attention meditation practices may be fundamentally quite similar to other types of skill acquisition shown to induce neuroplasticity (Lazar et al., 2005; Pagnoni and Cekic, 2007). During the early stages of meditation training, an emphasis on sustained attention and an ability to focus attention on a single object such as the breath, is often the first and most difficult skill to develop for relatively novice practitioners (Brefczynski-Lewis et al., 2007). While numerous studies have implemented novel neurofeedback protocols for the purposes of investigating brain function and neuroplasticity, as well as training memory and attention (Sitaram et al., 2017), an inspiring application of neurofeedback may be to help offer alternatives for individuals who may benefit from the direct engagement and feedback during meditation practice. Various methods of delivering neuro and biofeedback across a range of modalities have been shown to significantly enhance learning processes. Neurofeedback paradigms developed for providing feedback reflecting targeted measures of attentional focus during meditation may bridge access to a broader audience and to those more easily discouraged due to the initial difficulties encountered with meditation practices, and may also benefit more experienced meditators interested in evolving their practice or learning alternative meditative techniques.

We therefore designed a novel double-blind 8-day closed-loop neurofeedback protocol to adaptively train and up-regulate FMθ in meditation novice participants while implementing several key strategies derived from the core methods used in focused-attention meditation practice. Given that the efficacy of neurofeedback protocols are thought to be dose-dependent (i.e., the number of sessions across time), and that lengthy protocols are often unsuccessful due to participant drop-out, cumbersome EEG recordings, and complex implementation and measurement techniques, we explored the plausibility that when coupled with focused-attention mediation based strategies, participants receiving the real-time adaptive neurofeedback (as compared to their age and gender matched active controls) would demonstrate an enhanced capacity for modulating FMθ after only eight 30-min neurofeedback sessions.

## Materials and Methods

### Participants

Twenty four right-handed healthy participants (12 women; mean age: 25; SD: 3) participated in the study. Participants were assigned to the experimental neurofeedback group (NF; *n* = 12, 6 women; mean age 25; SD: 3) or the active sham control group (Sham NF; *n* = 12, 6 women, mean age 25, SD: 3). 12 initial participants signed up for the 2-week study, after which point only interested participants who matched the age and gender of an enrolled participant were invited to participate in order to establish the age and gender matched pairs. One participant from each pair was then randomly assigned to either the feedback or sham group. The technician running the neurofeedback data collection was blind to which participants received real versus sham feedback – this was achieved by anonymizing the information about the type of feedback participants were getting from the script that was used to run the experiment. Participants assigned to the sham feedback group viewed a replay of the feedback previously recorded from the matched neurofeedback participant who received real feedback. This type of matching was done in order to normalize the visual statistics and potential influence of the visual stimulus. Participants received 10 euros per hour, were recruited through a university email list, were informed of the broader goals, protocol, schedule, and aims of the experiment, provided written consent, and had normal or corrected to normal vision. The protocol was approved by the Comite de Protection des Personnes (CPP) de Toulouse II Sud-ouest (protocol 10009 4/12/2015).

### Experimental Protocol

**FIGURE 1:**
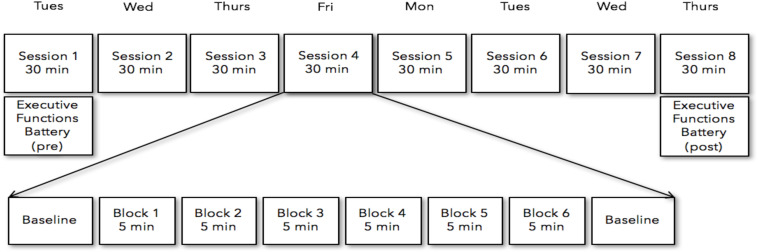
Participants received either the neurofeedback or sham-neurofeedback training over the course of eight training from Tuesday to Friday in the first week, and from Monday to Thursday in the second week. Each Neurofeedback training session consisted of six five-min training blocks, separated by short 2–6 min breaks. The battery of tasks measuring executive functions were collected pre-neurofeedback on the first session and post-neurofeedback on the last session.

Participants received either the neurofeedback or sham-neurofeedback training over the course of eight training sessions within two consecutive weeks. Neurofeedback training sessions were conducted from Tuesday to Friday in the first week, and from Monday to Thursday in the second week (Figure 1). On the first and last days of neurofeedback training participants completed the Executive Functioning Battery (EF battery; ∼40 min). The EF battery was collected pre-neurofeedback on the first session and post-neurofeedback on the last session. Each Neurofeedback training session consisted of six 5-min training blocks, separated by short 2–3 min breaks. This was done to assess the comparability of both subject groups with respect to motivation, commitment and perceived training difficulty. The length of the five-min training were implemented to prevent concentration declines (Enriquez-Geppert et al., 2014). Each subject came into the lab for the Neurofeedback sessions at the same time of day as their initial recording throughout the experiment. This was to ensure that a full 24 h had passed between the previous session and that this time duration was standardized across participants (i.e., a participant recorded at 10am the first day returned throughout the experiment at 10 a.m.).

### EEG Recordings

Data were collected using a 64-channels Biosemi system and a Biosemi 10-20 head cap montage at, 2048 Hz sampling rate for the first and last day of the protocol. We used the BIOSEMI ActiView software to ensure that all electrodes were kept within an offset of 15 (offset is the Biosemi method for measuring impedance). Days 1 and 8 included the pre and post executive functioning assessments in addition to the first and last session of Neurofeedback. For the remaining Neurofeedback sessions (Days 2–7) EEG activity were recorded from 8 electrodes locations Fpz, FZ, F7, F8, Cz, P7, P8, Oz using the external input EXG1 to EXG8 of the BIOSEMI amplifier. For the adaptive neurofeedback, EEG data were acquired using Lab Streaming Layer Software (LSL), and the visual stimuli were generated and presented using PsychToolbox in Matlab (v3.0.8) (Brainard, 1997) both running simultaneously on Windows 7 desktop workstation. EEG data were saved by both the Matlab script in.mat file and LabRecorder in.xdf files. The collection of Matlab scripts used to run the experiment is available on GitHub^1^. Synchronization for offline processing between stimulus timing from the Matlab psychophysics toolbox script and EEG signal was performed using an ADR101 board (Ontrak Control Systems Inc.) that converted serial input to parallel output sent to the Biosemi amplifier. This synchronization signal was used in offline processing to check the timing of the real time EEG streaming from LSL.

### Neurofeedback Training and Its Implementation

**FIGURE 2:**
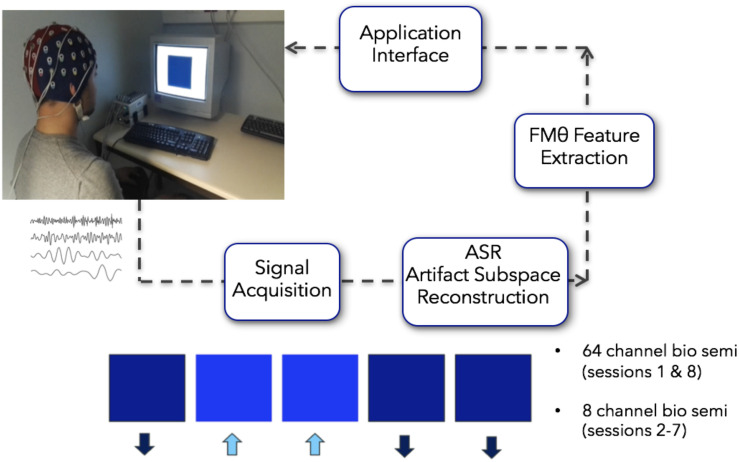
Closed loop Neurofeedback protocol starting from the acquisition of the signal to the automated ASR artifact rejection, to the FMθ extraction to the interface. When recording more than 8 channels (first and last sessions recorded 64 channels), the 8 channels Fpz, FZ, F7, F8, Cz, P7, P8, Oz were extracted from the data – although only these 8 channels were collected on all recording days, on the first day and last recording day 64 channels were recorded. Images in this figure were obtained with written consent.

All of the participants recruited for the study reported no previous meditation practice. Prior to the initial neurofeedback session, all participants reviewed a set of instructions which included specific strategies for how to engage with and modulate the neurofeedback with the central strategic element centering on breath focus. These instructions were to either (1) focus their attention on the physical sensations that occur with inhalation and exhalation of air moving in the nostrils, (2) focus their attention on the physical sensations associated with of the rising (inhalation) and falling (exhalation) movement of the abdomen, or (3) they could silently count each breath cycle (inhalation + exhalation = 1) up to 10 and repeat. Subjects were also instructed to bring their attention back to their breath (implementing their preferred strategy) in a non-judgmental manner if and when they noticed their mind-wandering, and to use whichever strategy felt most effective in the moment. Visual feedback of their ongoing EEG FMθ activity was given by means of a colored square (Figure 2). Depending on the FMθ amplitude, the color of the square changed in its gradient (relative to the baseline measurement) from light blue when FMθ amplitude was enhanced, to black when it was attenuated. Participants were further informed to use whichever strategy would favor a highly saturated and prolonged blue-coloration of the square (reflecting an increase of FMθ from baseline). Participants assigned to the neurofeedback group received real-time feedback of their own brain activity, while the sham-neurofeedback group received a playback of the feedback of the matched neurofeedback group participant (see Supplementary Material for video of a feedback block). To ensure methodological validity of implementation, participants were asked whether they were able to successfully implement one of the specified strategies after completing their daily NFB. All 12 NFB participants reported successfully implementing one or more of these strategies within and across each session in order to increase FMθ amplitude relative to the amplitude during resting EEG, however, 8 of the 12 NFB-Sham participants reported a lack of coherence with their perceived successful implementation of the strategies and the feedback of the visual stimulus.

### Real-Time Data Processing of Neurofeedback

The FMθ neurofeedback was implemented using 8 channels (Fpz, FZ, F7, F8, Cz, P7, P8, and Oz). The first and last sessions recorded 64 channels that were used for analyses pertaining to the cognitive battery, however, the neurofeedback on these days was still implemented via the 8 channels specified above (these were extracted in real-time from the 64 channel montage). On days two through seven, 8 individual EEG channels were recorded. Analysis windows of 1 s duration with a 75% overlap were considered so the neurofeedback display would be updated 4 times per second. Upon acquisition, each data chunk (of 1/4 of a second) was down-sampled from 2048 to 256 Hz and high pass filtered at 0.5 Hz using minimum-phase FIR filter, preserving the state of the filters from one block to the next – using the flt_fir filter function of the BCILAB program (Kothe and Makeig, 2013). Data from the 8 channels was then average referenced.

**FIGURE 3:**
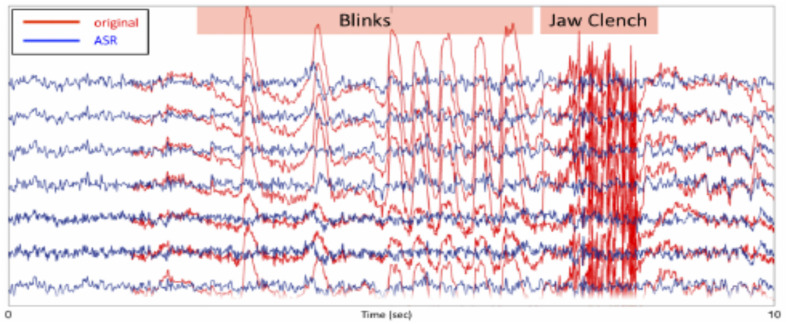
For artifact rejection, each day an initial EEG baseline was measured for 1 min (start baseline), followed by six training blocks of 5 min each (block 1–6). This initial baseline was used by the ASR artifact rejection algorithm in order to optimize the filtering all feedback sessions for the day using the asr_calibrate function (default parameters of the ASR algorithm were used; variance rejection cut off of 5; block size of 10 sample to calculate covariance matrix; window size of 500 ms and window overlap of 330 ms.

For artifact rejection, each day an initial EEG baseline was measured for 1 min (start baseline), followed by six training blocks of 5 min each (block 1–6; Figure 3). This initial baseline was used by the ASR artifact rejection algorithm in order to optimize the filtering all feedback sessions for the day using the asr_calibrate function (default parameters of the ASR algorithm were used; variance rejection cut off of 5; block size of 10 sample to calculate covariance matrix; window size of 500 ms and window overlap of 330 ms; Mullen et al., 2013). The filter was applied to the data using the function asr_process. Note that a bug in the program made it so that the state of the ASR filter was not propagated properly from one data block to the next, generating brief small signal discontinuities. This may have affected the quality of the real-time feedback – but did not affect *post hoc* data analysis for which we were able to apply compensatory measures to restore the signal continuity.

To calculate feedback, spectral power over a sliding 1 se window was calculated using the Fast Fourier-transform (FFT; after using a hamming tapering window) on channel Fz. Spectral power p was calculated at 4, 5, and 6 Hz by taking the square amplitude of the FFT at these frequencies, log transforming these values and subsequently averaging them. To provide a smooth visual feedback experience, the following procedure was applied every 250 ms and the feedback value f between 0 and 1 was calculated as follows. First an intermediate feedback value was calculated according to*f* = (*p*−*l*)/(*h*−*l*) where f is the feedback value, l is the lower edge of the dynamic range and h is the upper edge of the dynamic range. If *f* is lower than 0 then, it was capped to 0 and the lower edge was decreased *l* = *l*−(*h*−*l*)/30otherwise it was increased*l* = *l* + (*h*−*l*)/100. Similarly if *f* is larger than 1 then it was capped to 1, and the upper edge was increased *h* = *h* + (*h*−*l*)/30 otherwise it was increased by*h* = *h*−(*h*−*l*)/100. This procedure ensured that the dynamic range for the feedback value would adjust to participants’ theta changes over time. This yielded a final feedback value between 0 and 1. In addition any change to the feedback value larger than 5% compared to the previous value was capped at 5% in the direction of the change. This setup was chosen to provide the participants with a smooth appearance of the visual feedback by avoiding sudden jumps in the feedback colors. Note that this procedure was also run – although the display was disabled – during the 1 min preparatory baseline period which preceded the first feedback session which allowed the program to calculate an acceptable theta dynamic range for the onset of the first neurofeedback block. Dynamic range at the end of each of the 5 min block, was used as a starting point for the following 5 min blocks. At the end of the training session a second EEG baseline was measured (end baseline).

Neurofeedback stimuli were presented on a 17” DELL M781 mm CRT computer screen set to 75 Hz with a resolution of 800 × 600. The feedback value from 0 to 1 controlled the color of a blue square 400 × 400 centered on a screen. Feedback 0 was black (RGB #000000) and feedback 1 was a highly saturated blue (RGB #0000FF) with intermediate feedback values yielding a hue of blue proportional to the feedback in the RGB color space (for example feedback 0.5 was RGB #000080). The color of the square was updated 4 times per second (see Supplementary Video 1).

### Neurofeedback Offline Data Processing

All neurofeedback parameters were saved after each session and used to assess the efficacy of neurofeedback training. The 64-channel sessions at the beginning for the first and final sessions were concatenated and further processed. EEG data was down-sampled from 2048 to 256 Hz and processed offline in MATLAB (MathWorks, Inc.) and EEGLAB (Delorme and Makeig, 2004) in order to compute time frequency and spectral differences. All neurofeedback data were filtered and pre-processed online. Additionally, we automatically removed 5 temporal electrodes from the 64-channel electrode montage (PO2, PO3, POz, P2, PO4) that appeared noisy across a majority of participants, with a total average of 6 per electrodes per subject using EEGLAB pop_rejchan function,(this removes electrodes which had a kurtosis larger than 5 standard deviation compared to the ensemble of all electrodes). We also removed portions of data with high frequency content which spectrum was larger than 10 dB (compared to the mean power in the whole recording) in the 20–40 Hz frequency band over 4 contiguous windows of 0.5 s (pop_rejcont function of EEGLAB) to potentially remove artifacts that were not removed online.

### Statistical Analyses: Training Effects on Frequency Amplitudes

For the analyses of neurofeedback success, the relative change in FMθ amplitude across all six neurofeedback blocks for each of the eight sessions was quantified as change in microvolts and percent relative to the corresponding values of the first training session/day. In addition applying this calculation to theta activity, to investigate the specificity of training success this calculation was also performed for alpha and beta activity. Resting EEG for each session/day (1–8) was calculated as the mean of the start and end baseline measurements relative to FMθ amplitude observed during the baseline measurements of the first session/day. Training effects were analyzed by a repeated-measures ANOVA with the factors session (1–8) and group (neurofeedback vs. sham) for training amplitude (I). To investigate the course of FMθ amplitude increase during training, a regression line was fitted for each subject (II). To test if gradients were different between groups (neurofeedback vs. sham) a one-tailed independent-samples *t*-test was calculated for the slope and the intercept (III). Lastly, training effects on resting EEG were analyzed with a repeated-measures ANOVA with factors session (1–8) and group (neurofeedback vs. sham) for (IV). In cases of sphericity violations, Greenhouse–Geisser corrections were performed; corrected p-values as well as ε-values are reported.

### Statistical Analyses: Dynamical Changes Within Neurofeedback Sessions

A further method to identify changes due to neurofeedback is the analysis of changes within sessions compared to the baseline measurements (e.g., Vernon et al., 2009). Thus, training amplitude for each experimental block was extracted and averaged across all sessions (start baseline, blocks 1 through 6, end baseline) for FMθ, alpha, and beta frequencies relative to the amplitude observed during the first start baseline as change in percent. Effects were analyzed by a repeated-measures ANOVA with factors block (start baseline, blocks 1 through 6, end baseline) and group (neurofeedback vs. sham). Custom scripts under R were used as well the Statistica software to perform statistical analysis.

### Executive Functioning Tasks

One the first and last day, participants performed and executive functioning battery consisting of a n-back task, SART, and a Local Global task. We additionally collected participants’ anatomical and functional MRI before and after the neurofeedback protocol, however the results will be reported separately. For all stimulus presentations, we used a desktop computer running the Matlab Psychophysics toolbox (v3.0.8) under Windows 7 operating systems. Stimuli were presented on a 17” DELL M781 mm CRT computer screen set to 75 Hz with a resolution of 800 × 600.

#### N-back Task

**FIGURE 4:**
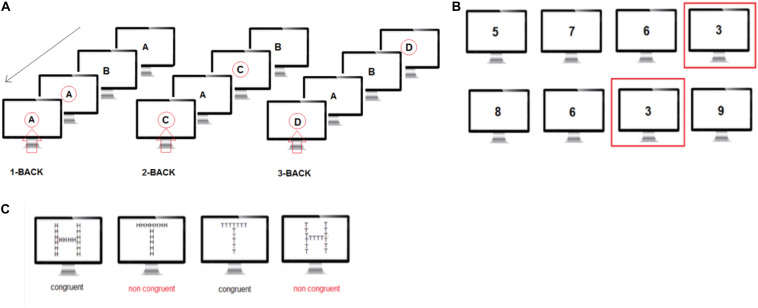
**(A)** N-back task: visual illustration of the three levels of the n-back task. The red arrow indicates when the subject has been instructed to press the space key. **(B)** The Sustained Attention to response task (SART) is designed to measure a person’s ability to withhold responses to infrequent and unpredictable stimuli during a period of rapid and rhythmic responding to frequent stimuli. **(C)** The local-global task measures the ability to focus attention on a specific feature of a stimulus, either global or local, while resisting distraction from other features and is thought to be a relatively broad measure of conflict detection.

Participants performed a visual sequential letter n-back working memory task, with memory load ranging from 1-back to 3-back. The visual stimuli consisted of a sequence of 4 letters (A,B,C,D) presented black on a gray background. The participants observed stimuli on a visual display and responded using the spacebar on a keyboard. In the 1-back condition, the target was any letter identical to the trial immediately preceding one (i.e., one- back). In the 2-back condition, the target was any letter that had been presented two trials back, and in the 3-back condition, the target was any letter presented three trials back. In this way, working memory load varied from 1 to 3 items. Stimuli were presented on the screen for a duration of 1 s, after which a fixation cross was presented for 500 ms. Participants responded to each stimulus by pressing the spacebar with their right hand upon target presentation. If no spacebar was pressed within 1500 ms of the stimulus presentation, a new stimulus was presented. Reaction times to each response were recorded. Each n-back condition (1, 2, and 3-back) consisted of the presentation of 280 stimuli selected randomly in the 4-letter pool (Figure 4A).

#### SART Task

The Sustained Attention to response task (SART) is designed to measure a person’s ability to withhold responses to infrequent and unpredictable stimuli during a period of rapid and rhythmic responding to frequent stimuli. Performance on the task is measured in participants’ ability to self-sustain mindful and conscious processing of stimuli whose repetitive, non-arousing qualities would otherwise lead to habituation and distraction to other stimuli, and is proved to be a sensitive measure of vigilance. Participants were presented with a series of single numerical digits (randomly selected from 0 to 9 – the same digit could not be presented twice in a row), and instructed to press the spacebar for each digit, except for when presented with the digit 3. Each number was presented for 400 ms in white on a gray background and took about 1/5 of the screen height. The inter-stimulus interval was 2 s irrespective of the button press and a fixation cross was present at all times except when the digits were presented. Participants performed the SART for ∼10 min corresponding to 250 digit presentations (Figure 4B).

#### Local Global Task

The local-global task measures the ability to focus attention on a specific feature of a stimulus, either global or local, while resisting distraction from other features and is thought to be a relatively broad measure of conflict detection. In the local-global task, participants were shown large letters (H and T) on a computer screen. The large letters were made up of an aggregate of small letters that could be congruent (large H made of small Hs or large T made of small Ts) or incongruent (large H made of small T’s or large T made of small Hs) with respect to the large letter. The small letters were 0.8 cm high and the large letters were 8 cm high on the computer screen. A fixation cross was present at all times except when the stimulus letters were presented. Letters were shown on the computer screen until the subject responded. After each subject’s response, there was a delay of 1 s before the next stimulus was presented. Before each sequence of letters, instructions were shown on a computer screen indicating to participants whether they should respond to the presence of small (local condition) or large (global condition) letters. We instructed participants to categorize specifically large letters or small letters and to press the letter H or T on the computer keyboard to indicate their choice. Participants performed a total of 200 trials in 4 sessions of 50 trials each. In sessions 1 and 3, participants were instructed to focus on large letters and in sessions 2 and 4 they were instructed to focus on small letters (Figure 4C).

### Executive Functioning Data Processing

Data processing was performed in Matlab (Mathworks, Inc.) and EEGLAB (Delorme and Makeig, 2004). The raw EEG data was average referenced and down-sampled from 2048 to 256 Hz. A high-pass filter at 1 Hz using an elliptical non-linear filter (IIR; transition bandwidth of 0.7 Hz and order of 6) was applied, and the data was then average referenced. Extended Infomax Independent Component Analysis (ICA) was applied to the data (Delorme et al., 2007). ICA components for eye blink, lateral eye movements, and temporal muscle noise were identified and subtracted from the data by the visual inspection of both the component scalp topography and power spectrum distributions. Between 1 and 5 artifactual components were removed for each subject. Bad electrodes (0–15 per subject, an average of 6 per subject) and bad epochs containing paroxysmal activity were manually removed from the data.

## Results

### Statistical Results: Neurofeedback Effects on Amplitudes (Neurofeedback vs. Sham)

**FIGURE 5:**
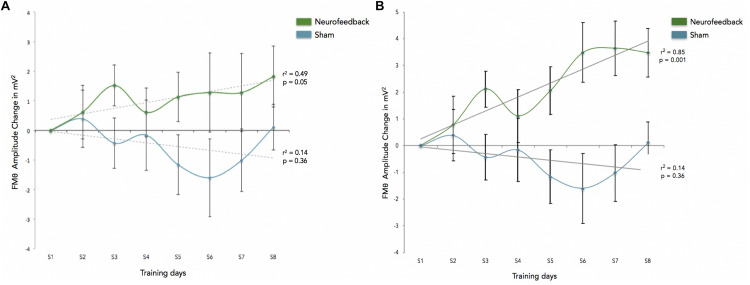
**(A)** This graph shows the enhancement across sessions, and reflects FMθ amplitude percent change for the mean of theta power for the Neurofeedback group (green) and the sham group (blue) across each training session (S1–S8) as averaged over all corresponding blocks (blocks 1–6) as compared to the first session (S1). Baseline amplitude changes are reflected by the dotted lines for each group respectively, and are show for the training relative to the first baseline measurements. Error bars indicated the standard error of the mean. **(B)** This graph shows the enhancement across sessions for participants identified as responders to the adaptive neurofeedback protocol (three non-responders have been removed from the analyses). Non-responders are identified as individuals whose daily scores were three or more standard deviations from the mean. As in this figure shows the enhancement across sessions, and reflects FMθ amplitude percent change for the mean of theta power for the adaptive neurofeedback group (green) and the sham group (blue) across each training session (S1–S8) as averaged over all corresponding blocks (blocks 1–6) as compared to the first session (S1). Error bars indicate the standard error of the mean.

**FIGURE 6:**
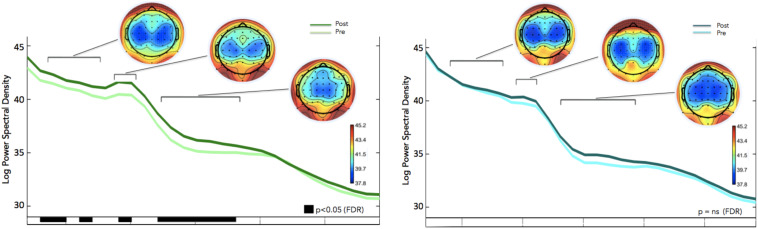
Frequency Spectra for the Neurofeedback group (top, green) and sham group (bottom, blue) showing differences in averaged spectral power between pre (session 1) and post (session 8) for electrode Fz (feedback location). Significant differences in multiple frequency bands were observed for the Feedback group (*p* < 0.05, corrected for multiple comparisons; *p*-values are reflected as black bars above the x-axis). No significant differences were observed in the sham group.

We calculated how theta power varied across groups (neurofeedback vs. sham), by averaging the EEG activity across the sessions (1–6) for each day (days 1–8) and entered the three factors (group, day and session) into a General Linear Model analysis (GLM). We also included subject as factor that was hierarchically nested within Groups (because different groups contain different participants). Including or not including participants in the GLM returned similar results although including participants tended to increase significance. Notice that Group is the only categorical variable (subject is also a categorical variable but since it is nested within groups it is not possible to calculate the interaction with Group). Sessions and days were both considered continuous variables. For all GLM fitting parameters we used the default of the Statistica software. We observed significant difference in theta between groups, and significant differences in theta between sessions. FMθ power was larger for the neurofeedback group [44.62 equivalent dB (10^∗^log_10_(mV^2^)] compared to the sham group [44.35 equivalent dB (10^∗^log_10_(mV^2^]). Neurofeedback training effects and baseline amplitudes are shown (Figure 5A) for both groups. Statistical analyses using R software were used to perform a Pearson correlation test which serves as a measure of linear correlation between our two groups (sham group: *r*^2^ = 0.14, *t* = -0.99, *df* = 6, *p* = 0.36, neurofeedback group: *r*^2^ = 0.49, *t* = 2.42, *df* = 6, *p* = 0.05; Figure 5A). Our results show a significant correlation for the neurofeedback group, while no significant relationship between the evolutions of FMθ for the sham group was observed (neurofeedback group: *r*^2^ = 0.49, *t* = 2.42, *df* = 6, *p* = 0.05; sham group: *r*^2^ = 0.14, *t* = -0.99, *df* = 6, *p* = 0.36; Figure 5A). In line with previous neurofeedback research suggesting that approximately 25% of participants who receive neurofeedback do not respond to neurofeedback (see section “Discussion”; Zoefel et al., 2011), we identified 25% of real-NFB participants (*n* = 3) whose total power values were greater than 3 standard deviations from the mean. These findings align with previous observations made in earlier neurofeedback studies. After excluding non-responders from the NFB group, a more robust trend was observed at the group level (sham group: *r*^2^ = 0.14, *df* = 6, *p* = 0.36, neurofeedback group: *r*^2^ = 0.85, *df* = 6, *p* = 0.001, Figure 5B). The regularity of the shape of the curve and of the growth during the sessions in the neurofeedback group notably contrasts with the control group which presents a more heterogeneous and chaotic activity. Also in line with previous neurofeedback studies (Zoefel et al., 2011), we observed significant effects in the broader EEG spectra over the training electrode site Fz. Permutation statistics conducted on the EEG spectral power at Fz revealed significant differences for FMθ (3.5–6.5 Hz, *p* < 0.05), low alpha (9–10 Hz, *p* < 0.05) and beta frequencies (12–18 Hz, *p* < 0.05, Figure 6). No significant differences were observed in the sham group.

### Behavioral Results on Executive Functioning Tasks

**FIGURE 7:**
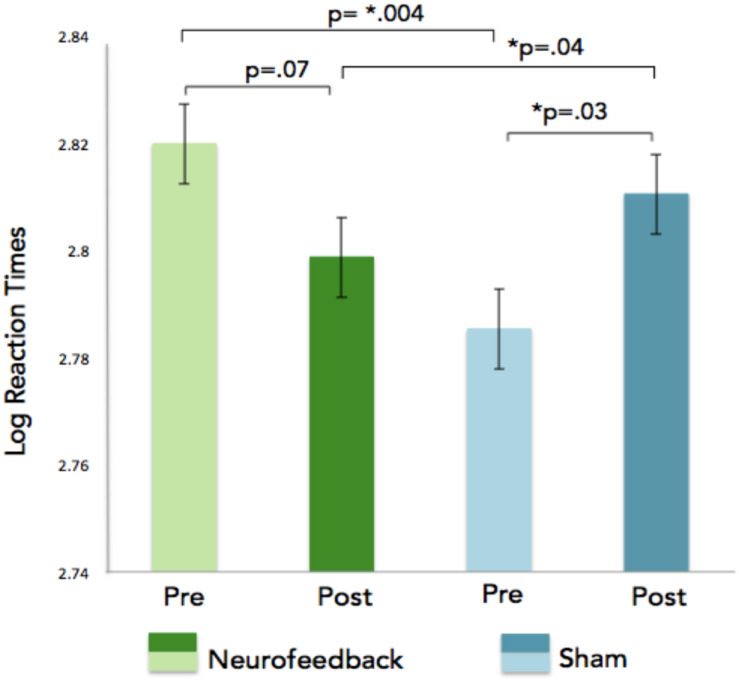
Log reaction times are indicated on the x-axis for the n-back task. The GLM revealed a significant pre/post neurofeedback training interaction showing faster reaction times for correct n-back trials in the neurofeedback group as compared to the sham feedback group after neurofeedback training.

GLM analyses were performed on the number of correct responses and reaction times. We choose to use this solution (rather than a collection of *t*-tests based on group compared with a variety of sub conditions) to avoid the problem of having to correct for multiple comparisons. In addition, the GLM allows capturing all the subtleties of the data and provides an overarching view of the broader effects at once without having to run multiple analyses. As was done for the neurofeedback data, each subject was added as a factor hierarchically nested within groups (removing these factors did not dramatically affect the results). GLM analyses on the n-back task reaction times revealed a significant interaction effect for the session (pre vs. post), showing faster reaction times for correct n-back trials in the neurofeedback group as compared to the sham feedback group after neurofeedback training (Figure 7). For correct responses, significant effects were observed for the condition (1, 2, 3 back), session, and response (type of response), as expected given that there are more correct responses for 1-back than for 2-back and more correct responses for 2 back than for 3 back (Table 1). Condition by response is significant indicating an effect of the condition (1, 2, or 3 back) on the number of hit and true negative. Analysis of the SART and the local-global tasks pre and post neurofeedback yielded no statistically significant results.

**TABLE 1:** Statistical results of the GLM analysis for the number of correct responses.

Nback-correct response	*F*	*p*	DF
Intercept	9411.69	0.0000	1
Group	0.36	0.5516	1
Condition	127.36	0.0000	2
Session	8.60	0.0037	1
Response	900.74	0.0000	2
Group∗Condition	0.84	0.4319	2
Group∗Session	0.14	0.7136	1
Condition∗Session	1.68	0.1891	2
Group∗Response	2.00	0.1582	1
Condition∗Response	17.82	0.0000	2
Session∗ Response	3.74	0.0544	1
Group∗Condition∗Session	0.05	0.9542	2
Group∗Condition∗Response	2.43	0.0904	2
Group∗Section∗Response	0.39	0.5356	1
Condition∗Section∗Response	0.19	0.8286	2
2∗*3*∗*4*∗5	1.34	0.2638	2
Subject (Group)	2.78	0.0001	22
Error			242

*Significant effects were observed for the condition (1, 2, 3 back), session, and response (type of response), as expected given that there are more correct responses for 1-back than for 2-back and more correct responses for 2 back than for 3 back.*

### EEG Activity for the Executive Functioning Tasks

**FIGURE 8:**
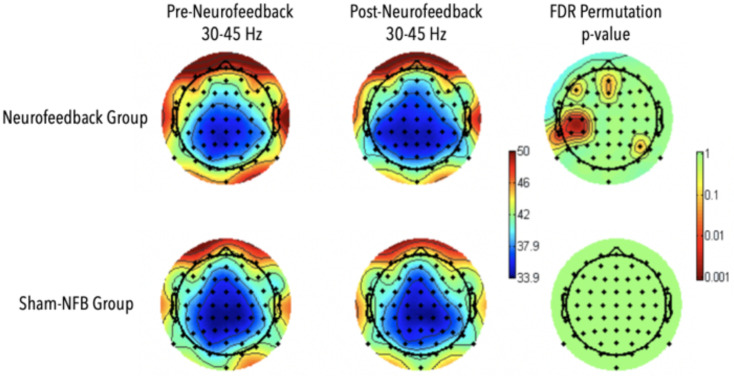
Results for Adaptive Neurofeedback (top) and sham (bottom) groups for gamma-power during the 2-back task, before (left) and after (middle) the adaptive neurofeedback sessions. The color bar on the right represents the statistical significance of the difference pre and post neurofeedback.

A significant increase in gamma power in the frontal midline as well as areas spanning the left temporal parietal areas were observed during the N-2 back task for the participants who received NFB (*p* < 0.01; Figure 8). No significant differences were observed in the sham group, and no significant interaction effects were observed between neurofeedback and sham participants. Differences in EEG activity and behavioral measures for the SART and local-global tasks were absent.

## Discussion

The findings from this study suggest that it is possible to train and reinforce the networks generating FMθ activity through a neurofeedback training protocol in which participants applied focused-attention meditation techniques. Participants in the neurofeedback group showed a significant increase in FMθ activity across sessions 1–8 as compared to active controls. To our knowledge this is one of the first studies to test the feasibility of neurofeedback training based on the implementation of meditation strategies, as well as for a specific frequency and location based on findings from advanced meditation practitioners. Given that the all participants reported the successful application of at least one of the meditation strategies (all of which facilitated the same core focus of attention to the breath) as a means of modulating the neurofeedback, the significant increase in FMθ observed across sessions serves as measure of validation regarding the nature of our novel meditation-FMθ combined feedback protocol. Differences in behavioral outcomes as observed by significantly faster reaction times on correct 2-back trials in the NFB training group compared to the active controls after only eight 30-min sessions serves as an additional indicator of neurofeedback training validation. We additionally recorded both functional and structural MRI (data will be presented in a separate manuscript) data to serve as a further means of neurofeedback protocol validation.

### Optimizing Neurofeedback Protocols and Identifying Non-responders

Our study showed successful modulation of FMθ training within only eight sessions (days) of neurofeedback training through the use of focused-attention meditation strategies. Additionally, we addressed several historically unspecified effects such implementing a reliable control group, and strictly controlled time lags between training blocks and the eight neurofeedback sessions. The percentage change in spectral EEG power due to neurofeedback training was relatively low in our task and may be due to variety number of reasons, including difficulty with the implementation of the meditation based strategies, frustration due to perceived feedback parameters, or lack of motivation. In terms of the percentage of positive neurofeedback reward that should be provided, various reports indicate that this percentage remains under debate (Sitaram et al., 2017).

Increased temporal lags within training and between training sessions has been shown to enhance training gains (Ebbinghaus, 1964) and are based on two time scales: gaps between days, and the gaps within a given neurofeedback session. The exploration of training effects of different long-lasting training lags (from minutes up to several days) on a systematic level is important concerning at least to two aspects: with respect to the investigation of neuronal correlates and in regard to find the optimal repetition interval for neurofeedback. While here we implemented a strict protocol with 24 h passing between each session (excluding the two day break over the weekend) and 2–3 min breaks between the 5 min sessions, alternative schedules and training gaps should be explored in future research. Additionally, the percentage of feedback provided to participants is thought to significantly influence learning. While 80% positive feedback has been considered to be too high for optimal learning (Arns et al., 2014), too low of a percentage prevents the subjective experience of feeling in control. It has been suggested that a moderate amount (50%) of positive feedback may increase the generation of the desired behavior during neurofeedback and thus transfer more easily into daily life.

A general explanation for non-responders in the neurofeedback literature is the use of ineffective strategies, or strategies unrelated to the cognitive processes involved in the generation and modulation of the frequency of interest. Non-responders may have failed to modify the interface of neurofeedback and consequent learning during the sessions by not integrating the feedback needed for the modulation, however, a more likely explanations may involve individual differences impedance, abnormal baseline measurements, as well as differences in brain structure and fissuration, which has been linked to the predictability of neurofeedback training success. These hypothesis will be explored in the subsequent publication of the structural and functional MRI data.

The observed differences in reaction times on correct 2-back trials in addition to a corresponding increase in gamma activity in the NFB training group compared to the active controls after only eight 30-min sessions serves as an additional indicator of neurofeedback training validation. These behavioral and corresponding EEG findings indicate that perhaps the meditation-FMθ combined feedback protocol targeted and reinforced neural mechanisms specific to the n-back task such as goal directed behavioral and working memory (Gajewski et al., 2018) as they both require the active maintenance of the goal state, which should arguably be impenetrable to distractions viewed as irrelevant to the goal state, in addition to high demand attentional flexibility vs. stability (Figure 8). Furthermore, gamma activity observed in the participants who received meditation-FMθ combined feedback corresponded with the visual word form area, a left inferior temporal region specifically devoted to the accurate processing of letter strings such as those presented in the n-back task, in addition to the frontal mid-line sites that were the target of the neurofeedback training. Future research should further investigate the nuanced mechanistic differences and generalizability of behavioral measures that result from varying neurofeedback protocols.

### Trainability and the Neuroanatomy of FMθ

Research investigating the intraindividual effects of cognitive and behavioral trainings have found that regional differences in brain structure have been linked to predictability of training effectiveness in complex cognitive tasks and language learning (Basak et al., 2011; Loui et al., 2011; Wong et al., 2012; Ericsson et al., 2018). Enriquez-Geppert et al. (2014) found that inter-individual variations in MCC structure and cortical fissuration predicted the success of FM neurofeedback training. Is known that the MCC and dorsal ACC exhibit significant structural variability due to the large presence of fissures and convolutions, and that these variations have been linked to differences in executive functions (Huster et al., 2009, 2011). Enriquez-Geppert et al. (2013) hypothesized that the neuroanatomical structure, and high concentration of convolutions could very well play a role in the differences of results observed during the reinforcement of FMθ neurofeedback. They found that increases in FMθ power measured during the initial training sessions predicted the success of the FMθ increase across the eight sessions of training, while pre-existing inter-individual differences in the morphology of the right MCC, as well as higher white matter concentration of the right and larger volumes of the left cingulate bundle were associated with stronger FMθ enhancement during initial training success. Furthermore, large intra-individual differences, including the presence of additional sulci in the ACC region in approximately half the population, have been observed in the macroscopic anatomy of the cingulate and present an obstacle in resolving the subtle details in the regions functional organization (Shackman et al., 2011). We will explore our anatomical and functional MRI data in a future report.

### FMθ Feedback Implementation

Several neurofeedback studies have already attempted to target the theta, alpha, beta or sensorimotor rhythms in an attempt to train attention (Kaiser and Othmer, 2000; Egner and Gruzelier, 2004; Arns et al., 2009), memory (Nan et al., 2012; Wang and Hsieh, 2013; Staufenbiel et al., 2014), and executive functions (Hanslmayr et al., 2005; Zoefel et al., 2011). Whereas previous protocols have instructed participants to engage in specific cognitive strategies to train FMθ (Enriquez-Geppert et al., 2014), in the current paradigm participants were instructed to focus on their breath, engage in breath counting, or a relaxed visual focus of attention on the visual stimulus as methods for engaging with the feedback.

While the only previous research study investigating FMθ neurofeedback used individualized peaks of theta (∼5 Hz) as determined by a series of cognitive tasks (Enriquez-Geppert et al., 2013), we implemented our feedback based on a 4–7 Hz average for several reasons. Recent research has highlighted the existence of several different generators of theta in the frontal cortex, potentially all of which contribute to broader cognitive control (Cavanagh and Frank, 2014), with respectively different corresponding FMθ peaks and underlying neural microcircuitry (Cohen, 2014). Since it may the case that the frontal theta observed during meditation reflects a broad form of cognitive monitoring and control, it is our assumption that by choosing a specific peak frequency participants may not find an appropriate strategy that corresponds to an accumulative increase in FMθ power. Given that FMθ power may reflect several different temporal and topographic generators independently contributing to the FMθ power measured by EEG over the frontocentral cortex (Fz, FCz, Cz), we chose to provide feedback based on a broader theta range, as the cognitive control trained during meditation may reflect the cooperation of several different neural generators across the frontal cortex, each with potentially differing preferred spectral theta peaks. While a majority of neurofeedback studies encourage participants to engage in different types of strategies such as mental operations, emotions, imagination, memories, and thoughts of movements, and strategies that resemble a form of intentional mind-wandering, here we chose to provide a limited number of strategies that draw on the fundamental teachings of focused-attention meditation practice.

### Inclusion of an Active Control Group

Throughout the history of neurofeedback research, one of the central points of criticism has been the omission of appropriate control groups (Gruzelier and Egner, 2005; Gevensleben et al., 2009). While many protocols control for practice and repetition effects through the use of passive control groups, additional effects may significantly influence the success of training such as expectancy and placebo effects, both of which have been linked to improvements in clinical drug study outcomes (e.g., Price et al., 2008). Another potentially mediating factor in neurofeedback studies may be the exposure to the visual feedback itself. Similar to previous findings on the effects of sham-neurofeedback, we also observed some trending but non-significant changes in the EEG in the sham group, however in the case of our experiment individuals also engaged in focused-attention meditation practices across the eight sessions. The results from this study underline the importance for adequately controlling not only for repetition-related but also for such non-specific effects.

### Neuronal Consolidation

In general, two types of neuronal consolidation can be distinguished: synaptic vs. system consolidation. After the first hours of training, synaptic plasticity takes place including the formation of new connections and the restructuring of existing ones (Dudai, 2004). Since research investigating the differential effects of training lags is an important step for optimizing training protocols, the current protocol implemented mandatory 2–3 min breaks between each 5 min neurofeedback session. Additionally, participants we’re required to arrive for each neurofeedback session at the same time every day in order to assure that 24 h had passed between each day of training. To our knowledge, this is the first study to implement such a rigorously timed neurofeedback protocol in an effort to control for biases due to the varying daily physiological cycles. Sleep also significantly contributes to consolidation as during sleep a so-called “replay” of memory might take place (Huber et al., 2004). Spontaneous low-frequency neural oscillations, rhythmic spike bursts, and spike trains fired by thalamic and neocortical neurons that occur during heightened vigilance have previously been linked to the mechanisms underlying neuronal plasticity. These mechanisms are very similar to those that characterize slow-wave sleep, suggesting that slow-wave sleep may function to consolidate memory traces acquired during wakefulness in corticothalamic networks (Steriade and Timofeev, 2003). System wide consolidation refers to the slow reorganization of neural circuitry, most likely reflecting the stabilization of the newly formed memories (Frankland and Bontempi, 2005).

Research suggests that the MCC is strongly interconnected to cortical and subcortical areas and plays a critical role of information integration during goal directed behaviors (Lezak et al., 2004), executive functioning, and may facilitate the mechanisms for general action monitoring, through the entrainment of spatially distal functional networks via FMθ signals during cognitive control (Cavanagh et al., 2012; Cavanagh and Frank, 2014). Learning in the neocortex can be expressed by prediction errors that signal the need for network-wide adaptation, and are thought to enhance the future predictability and conserve cognitive resources (Dehaene et al., 1998; Friston, 2010). Increasing evidence would suggest that transient increases in FMθ reflect general surprise and detection of both endogenous and exogenous events (Brandmeyer, 2017) and may function to influence behavior through the enhanced sensory processing and the reallocation of attention (Mitchell et al., 2008). Thus, FMθ may function as a temporal template carrying higher information content signals such as gamma band activities via cross-frequency coupling. Given that long range neural inputs are likely to facilitate control over local inhibition and induce synchronous phase relationships (Buzsáki, 2004, 2010; Benchenane et al., 2011), a large variety of cognitive functions may be facilitated through the wide spread connections between the MFC and other brain areas (Fries, 2005; Phillips et al., 2014). Benchenane et al. (2011) propose that FMθ coherence may be due to an increase of dopamine modulated interneuron inhibition of pyramidal cells, after observing increased coherence in hippocampal-FMθ following the administration of dopamine in the prefrontal cortex of anesthetized rats.

Similar to the findings of Buzsáki (2004) in humans, Benchenane et al. (2011) found the activity in cell assemblies in the prefrontal cortex that emerged during increased FMθ coherence were replayed preferentially during subsequent sleep. Their interpretation was that coherence between the prefrontal cortex and hippocampus may lead to the synchronization of reward predicting activity in prefrontal networks, which are then tagged for later memory consolidation. MFC neurons differ from other cortical regions in terms of density, biophysical and anatomical properties and their specified theta band bursting properties combined with strong reciprocal excitatory (AMPA mediated) interconnections are thought to facilitate dopamine modulated short-term plasticity (Holroyd and Coles, 2002; Jocham and Ullsperger, 2009; Cohen, 2014). The neural mechanisms underlying such plastic changes in white matter involve the repeated activation of the specific neural pathways during learning in rats, primates and humans (Gibson et al., 2014; Wang and Young, 2014), and have also been evidenced by mental training methods such meditation (Tang and Posner, 2014). While recent findings now show that there are specific neuroanatomical criteria that can predict neurofeedback training success, it remains relatively unclear as whether these types of focal training protocols stimulate cerebral plasticity. FMθ modulation may be directly linked and dependent on the specific morphology of MCC neurons, features of white matter including increased bundle volumes, axonal density, or myelination which may help facilitate oscillatory FMθ interregional synchronization (Cohen, 2011).

### Limitations of Study

Interactions between outside factors such as sleep and exercise may highly impact the effectiveness of cognitive training protocols. Factors such as exercise have been shown to stimulate the new growth of stem cells in the hippocampus, with research showing enhanced optimization of new cellular structures when simultaneously paired with cognitive training measures (Van Praag et al., 2002; Shors et al., 2012; Shors, 2014). Studies comparing the effectiveness of mindfulness protocols that were paired or not paired with an exercise regimen found that participants who participated in the exercise intervention had highly significant improvements in various cognitive measures as compared to subjects who just received the mindfulness training (Shors, 2014). The direct relationship between neural plasticity and sleep has also been shown to play a key role in cognitive training effectiveness. Research has shown network wide reactivations of the (same) neuronal assemblies during sleep that have been recently involved in new and challenging environmental circumstances. These activations are presumably linked to the re-processing of memory traces during sleep. Post-training sleep deprivation has been found to significantly impair subsequent performance on various tasks, both in animals and humans. Additional research has shown an increase in REM sleep following training in several experimental conditions, and that this increased REM effect goes away after a given task has been mastered (Poe et al., 2000; Roozendaal, 2000). In the current study, we did not assess the number of hours of sleep for each preceding night and are therefore unable to explore whether or not the training gain effects were in any way correlated with sleep duration or quality. Additionally, we did not evaluate the motivation of subjects in a quantitative manner by which subjects could be objectively compared. Future studies should incorporate more refined neurophenomenological measures that address these factors in the experimental design.

### Neurofeedback as an Accompaniment to Meditation

One of the fundamental challenges that individuals experience when learning to meditate is the unceasing propensity of the mind to wander. Novice meditators may often find themselves discouraged after realizing that they had spent the majority of a meditation session unaware they had been mind-wandering or engaged in chronic thinking. This can be associated with strong emotional arousal during meditation practice, and may ultimately be detrimental to meditation practice and well-being (Delorme and Brandmeyer, 2019). Neurofeedback protocols that train the neural correlates associated with states of focused-attention (such as FMθ) may aid in the development of cognitive functions such as attention monitoring and metacognitive awareness of when the mind wanders, both of which are considered fundamental for meditation practice as well as for the broader regulation of attention. Interestingly, a large majority of neurofeedback protocols and meditation techniques aim to train attention and emotion regulation, for which cognitive engagement and attention monitoring are critical (Brandmeyer and Delorme, 2013). When an individual aims to improve their cognitive faculties so as to intentionally direct and actively sustain attention on an object of focus, they must develop an ability to incrementally adjust the amount of attention allocated to processing emotional stimuli by altering their judgments and expectations regarding emotional stimuli (Josipovic, 2010). Neurofeedback aided meditation may lessen the attention-grabbing power of mind wandering and spontaneous thought processes both during practice and in daily life, which may ultimately assist in deepening meditation practice. Attention and emotion regulation are central to both of these approaches, with the distinguishing elements being that meditation is self-regulated lacking any outside feedback, while neurofeedback is both machine aided and self-regulated, incorporating feedback elements that may serve to enhance or excel individual learning beyond that of self-guided meditation. Experimental designs that effectively assess refined first person accounts of neurofeedback protocols that experientially and directly correlate with changes in neural activity will greatly advance neurophenomenological approaches for studying and validating the neural correlates of meditative states and traits. Beyond meditation, self-regulated closed-loop neurofeedback paradigms will likely lead to the development of novel methodological approaches for the scientific investigation of embodied consciousness and the multidirectional interactions between the brain, body, and mind.

## Data Availability Statement

The datasets generated for this study are available at https://openneuro.org/datasets/ds001787 or by request. Please send inquiries to the corresponding author.

## Ethics Statement

The studies involving human participants were reviewed and approved by the Comités de Protection des Personnes, CPP, CNRS Toulouse. The patients/participants provided their written informed consent to participate in this study.

## Author Contributions

Both authors contributed to the article and approved the submitted version.

## Conflict of Interest

The authors declare that the research was conducted in the absence of any commercial or financial relationships that could be construed as a potential conflict of interest.

## Footnotes

1 https://github.com/arnodelorme/neurofeedbacklab
